# Spatiotemporal variations and environmental drivers of denitrifying anaerobic methane oxidizers in *Eriocheir sinensis* pond sediments

**DOI:** 10.3389/fmicb.2025.1679266

**Published:** 2025-09-25

**Authors:** Hongfei Zhang, Huimin Xu, Honghai Zou, Limin Fan, Xiangke Fan, Dandan Li, Longxiang Fang, Zhuping Liu, Hao Zheng, Liping Qiu, Shunlong Meng

**Affiliations:** ^1^Wuxi Fishery College, Nanjing Agricultural University, Wuxi, China; ^2^Freshwater Fisheries Research Center, Chinese Academy of Fishery Sciences, Scientific Observing and Experimental Station of Fishery Resources and Environment in the Lower Reaches of the Changjiang River, Wuxi, China; ^3^Jiangsu Province Fisheries Technology Promotion Center, Nanjing, China

**Keywords:** pond sediment, DAMO microorganisms, abundance, *Eriocheir sinensis*, environmental factors

## Abstract

Denitrifying anaerobic methane oxidation (DAMO), as a coupled carbon-nitrogen cycling process, facilitates methane oxidation while enabling inorganic nitrogen removal. Crab aquaculture pond sediments, being rich in organic matter and nitrogen, may serve as hotspots for active DAMO processes. However, the presence of DAMO-functional microorganisms in such environments remains unconfirmed. In this study, we employed quantitative real-time polymerase chain reaction (qPCR) to analyze the abundance variations of DAMO functional microorganisms in crab pond sediments across different temporal (May, September, December) and vertical (0–10 cm, 10–20 cm, 20–30 cm) scales. Combined with concurrent physicochemical parameter measurements and high-throughput sequencing, the spatiotemporal distribution patterns and environmental drivers of DAMO microbiota were investigated. The results indicated that DAMO bacteria and archaea coexisted in crab pond sediments, exhibiting significant spatiotemporal heterogeneity in microbial abundance. The copy number of bacterial *pmoA* gene ranged from 2.07 × 10^5^ to 1.89 × 10^7^ copies g^−1^ dry sediment, and archaeal *mcrA* gene ranged from 7.15 × 10^5^ to 1.16 × 10^8^ copies g^−1^ dry sediment. The abundance of both *pmoA* and *mcrA* genes peaked in December across all sampling timepoints, with their highest enrichment in the 10–20 cm sediment layer vertically, presumably due to the synergistic effect of a stable anaerobic environment, sufficient substrate supply, and moderate environmental parameters. Temperature, pH, and nitrite concentration were identified as key environmental factors regulating DAMO microbial abundance and spatial distribution. Furthermore, both microbial community composition and diversity indices displayed pronounced spatiotemporal variability, with seasonal variations exerting stronger impacts on community structure than vertical gradients. Notably, methane-metabolizing archaea exhibited higher species diversity than methane-metabolizing bacterial communities. This study systematically elucidates the ecological distribution patterns and environmental response mechanisms of DAMO-functional microorganisms in crab pond sediments, providing a theoretical framework for methane emission mitigation strategies in aquaculture systems based on DAMO processes.

## 1 Introduction

Aquaculture serves as a critical pillar for global food security and nutritional supply, emerging as an integral component in enhancing animal protein availability (Tadese et al., 2022). With advantages such as high productivity, high efficiency, and high resource utilization rate, aquaculture contributes 10.2% to China’s total agricultural output value and supports over 10 million direct and indirect jobs (Chen and Gao, 2023). This not only alleviates rural poverty but also stabilizes local people’s livelihoods. China’s production accounts for nearly 63% of the world’s total output in this sector (Zhang et al., 2025). The freshwater aquaculture of China predominantly adopts pond-based ecosystems, with an industrial scale spanning 2.6 million hectares (Hu et al., 2021). By 2020, the annual production of *Eriocheir sinensis*, a distinctive aquaculture species in China, surpassed 800,000 metric tons (Wang C. et al., 2019). Notably, owing to high water consumption and water pollution, this type of aquaculture model leads to the deterioration of the aquaculture water environment, and thus causes environmental issues such as reduced product quality, posing severe challenges to the development of aquaculture (Li et al., 2024). Research findings demonstrate that methane (CH_4_) fluxes in crab aquaculture systems exceed those in natural aquatic systems by 138%, thus rendering pond-based aquaculture a critical hotspot for greenhouse gas emissions (Dong et al., 2025). Such environmentally intensive production methods pose latent challenges to the industry’s sustainable development.

In crab pond aquaculture systems, low feed nutritional utilization efficiency combined with the specialized feeding behavior of *Eriocheir sinensis* leads to continuous accumulation of feed residues and excreta in sediments (Jia et al., 2023). These residues and excreta inherently contain elevated levels of carbon and nitrogen constituents. Their accumulation in sediments ultimately leads to the significant enrichment of carbon and nitrogen (Avnimelech, 1999; Bai et al., 2023). This environment provides abundant methane and nutritional substrates for denitrifying anaerobic methane oxidation (DAMO) microbial communities, thereby enhancing their metabolic activity and making these ponds potential hotspots for DAMO processes. DAMO represents a biochemical process where microorganisms oxidize methane under anaerobic conditions using NO₃^−^/NO₂^−^ as electron acceptors (Raghoebarsing et al., 2006). The DAMO process represents a coupled interaction between the carbon and nitrogen cycles, which is important for both methane mitigation and nitrogen removal. In the DAMO process, nitrate is reduced to nitrite by *Candidatus Methanoperedens nitroreducens* (*M. nitroreducens*), which is subsequently utilized by *Candidatus Methylomirabilis oxyfera* (*M. oxyfera*), and their synergistic cooperation enables coupled methane oxidation and denitrification (Ettwig et al., 2010; Shima et al., 2012; Haroon et al., 2013). However, there is no report that elucidates the existence of the DAMO process in the environment of *Eriocheir sinensis* ponds. Elucidating the ecological distribution of DAMO microorganisms in *Eriocheir sinensis* ponds not only deepens our theoretical understanding of carbon-nitrogen cycles in freshwater aquaculture systems, but also provides critical data to inform the development of sustainable aquaculture management strategies. This research is urgently needed to address the dual environmental challenges of greenhouse gas emissions and eutrophication in crab aquaculture systems adjacent to Taihu Lake, while also supporting the ecological protection goals of the entire Taihu Lake Basin.

As a crucial microbial pathway linking carbon and nitrogen cycles, the activity and ecological function of the DAMO process are comprehensively regulated by complex environmental factors. In the typical semi-artificial ecosystem of crab aquaculture ponds, DAMO microorganisms may be influenced by multidimensional drivers, including both biotic and abiotic factors. Further analysis of the key environmental variables that dominate the spatial and temporal dynamics of the DAMO process and the interactive effects among these factors provides potential regulatory targets for optimizing pond ecological management. In constructed wetlands, *M. oxyfera* affiliated bacterial communities exhibit pronounced seasonal variations, primarily influenced by temperature and nutrient availability (Xu Y. et al., 2018). Similarly, *M. oxyfera* abundance in the Zhoushan Archipelago displays seasonal fluctuations, which typically correlate with temperature regimes (Wang et al., 2017). Analogous seasonal variability is observed in DAMO archaeal populations (Xu et al., 2020). DAMO microorganisms are mesophilic, functioning optimally within a temperature range of 20–35 °C (He et al., 2015). Soil depth modulates DAMO microbial abundance; for instance, *M. oxyfera* density decreases with increasing sediment depth in Lake Biwa of Japan, whereas opposing trends are reported in natural freshwater wetlands (Kojima et al., 2012; Shen et al., 2015). Neutral pH conditions optimize DAMO microbial abundance and diversity, with physiological studies identifying *M. oxyfera*’s optimal pH at 7.60 (Zhang et al., 2018). As the only carbon source, high concentrations of methane increase the abundance of DAMO microorganisms but decrease their diversity, intensify colony competition, and accelerate community structure succession. Increased concentrations of ammonium nitrogen and nitrite nitrogen elevate *M. oxyfera* abundance, whereas total nitrogen and nitrate nitrogen concentrations alter microbial community diversity. Variations in nitrite and nitrate nitrogen levels simultaneously influence DAMO rates (Xu S. et al., 2018; Xu Y. et al., 2018). Nitrifying bacteria, anammox bacteria, and DAMO microorganisms exhibit interconnected yet competitive relationships, which dynamically shift with nitrogen speciation and concentration variations. Additionally, predation by aquatic organisms reduces both the abundance and diversity of DAMO microorganisms (Nie et al., 2021; Qu et al., 2022). In summary, the distribution of DAMO functional genes is subject to diverse driving factors, and analyzing the distribution patterns and driving mechanisms can provide a theoretical strategy for methane emission reduction from a DAMO perspective.

Therefore, it provides an important theoretical basis for microbial regulation of methane emission mitigation in aquaculture systems. In this study, stratified fixed-point sampling was conducted to collect spatiotemporally representative sediment samples. Absolute quantification of DAMO functional genes was performed using quantitative Polymerase Chain Reaction (qPCR). Physicochemical parameters of the sediments were determined to identify the key drivers regulating DAMO processes in the aquaculture pond system. Concurrently, 16S rRNA gene high-throughput sequencing was implemented to analyze bacterial and archaeal community compositions. The research aims to (1) elucidate the spatiotemporal distribution characteristics of DAMO functional genes associated with methane oxidation and denitrification processes and (2) identify the environmental drivers regulating their spatiotemporal dynamics.

## 2 Materials and methods

### 2.1 Research area

The experimental aquaculture site under investigation was located in Wujin County, Changzhou City, Jiangsu Province, China (119°51′03″E, 31°32′43″N), which exhibits the typical traits of *Eriocheir sinensis* cultivation within the Taihu Lake Basin (Supplementary Figure 1). This farm has been engaged in aquaculture for three years, and over these three years, there have been no changes to the cultured species, allowing it to consistently maintain a stable aquaculture structure. The aquaculture system consisted of 29.7 ha of regularly shaped polyculture ponds with an average water depth of 1.2 m, implementing a main-subsidiary integrated aquaculture model. Herein, *Eriocheir sinensis* served as the primary cultured species with a stocking density of 225 kg/ha, while *Palaemon carinatus* functioned as the complementary species undergoing ecological enhancement through polyculture synergism. The conventional culture cycle of *Eriocheir sinensis* extends from May to November. In May, as water temperature rises, the crab entered the formal culture stage. In September, the crabs reached commercial maturity and harvesting operations began. After the culture cycle ended in November, the ponds entered the preparation stage for drying. The comprehensive output per unit area in the system stabilized at 1650 kg/ha. Feeding was conducted using extruded pellet feed (corn-based) combined with trash fish, with two daily feedings at a rate of 45 kg/ha per application. During the aquaculture period, water was replenished to the ponds according to actual needs. No dredging operations were carried out during the aquaculture period, and the sediments accumulated naturally along with the aquaculture cycle. After the end of aquaculture each year, no active dredging interventions were conducted either.

### 2.2 Sample collection

To cover the entire aquaculture cycle, sediment samples were collected at three key time points in 2024, namely May (sediment–water interface temperature: 24.7 ± 0.3 °C), September (sediment–water interface temperature: 35.4 ± 0.5 °C), and December (sediment–water interface temperature: 14.2 ± 0.2 °C), which corresponded to the early aquaculture stage, mature aquaculture stage, and late aquaculture stage of *Eriocheir sinensis*, respectively. Sediment samples from 0 to 30 cm were collected in selected ponds using a cylindrical corer (Ф = 10 cm). To minimize the error, three pond sediment samples were randomly collected for parallel processing. Each core was systematically segmented into three profiles of 0–10, 10–20, and 20–30 cm into two portions in sterile polyethylene bags. The samples were temporarily stored in an insulated box maintained at 4 °C and transported to the laboratory for preservation within 8 h. One copy was stored at 4 °C for sediment physico-chemical property analyses, and the other at −20 °C for molecular biology analyses.

### 2.3 Measurement of sediment physical and chemical indicators

The collected sediment samples were freeze-dried, ground, and sieved through a 2 mm sieve to remove impurities. Temperature was measured at the sampling site using a thermometer. The sediment was mixed with water in a 1: 2.5 (w/v) ratio, and sediment pH was determined using a pH meter following equilibrium (Hou et al., 2013). The moisture content was determined by drying the samples at 105 °C and calculating the percentage mass loss. Loss on ignition (LOI) was assessed via high-temperature calcination in a muffle furnace at 550 °C, where the percentage mass loss after ashing corresponded to the organic matter content. Total nitrogen (TN) and total phosphorus (TP) were determined using the Kjeldahl method and alkali fusion-molybdenum antimony spectrophotometry, respectively. Sediment samples were extracted with 1 mol/L KCl solution to recover NH₄^+^, NO₂^−^, and NO₃^−^, followed by quantitative analysis of each ion in the filtrate (0.45 μm pore size syringe filter) via ultraviolet spectrophotometry (Zheng et al., 2014). SO₄^2−^ was determined via spectrophotometry after precipitation with BaCl₂ solution (Yang et al., 2013). Fe^2+^ was quantified using 1,10-phenanthroline spectrophotometry, followed by reduction of Fe^3+^ to Fe^2+^ with hydroxylamine hydrochloride solution to obtain total iron content. Fe^3+^ concentration was calculated as the difference between total iron and Fe^2+^ (Haese et al., 1997).

### 2.4 DNA extraction, PCR, and amplification sequencing

The PowerSoil® Pro DNA Kit (Qiagen GmbH, Munich, Germany) was utilized to isolate high-purity genomic DNA from 0.25 g of collected dry sediment samples following the manufacturer’s protocol. Sediment DNA concentration and mass were determined using a NanoDrop 2000 spectrophotometer (Thermo Fisher Scientific, Walthall, MA, United States). This study employed polymerase chain reaction (PCR) technology for amplification of the V3 − V4 hypervariable region of the 16S rRNA gene in both bacteria and archaea. The PCR amplification utilized two pairs of degenerate primers, 341F (5’-CCTAYGGGRBGCASCAG-3′) and 806R (5’-GGACTACNNGGGTATCTAAT-3′) for bacteria, Ar341F (5’-TCGTCAGCTGCCGTAAGGAA-3′) and Ar806R (5’-GGACTACNVGGGTWTCTAAT-3′) for archaea, in which a unique eight-base barcoding sequence was used for each sample (Vaksmaa et al., 2017). The PCR amplification system and thermal cycling parameters were established based on standardized protocols previously validated in literature (Chen et al., 2021). The amplified products were purified from a 2% agarose gel using the AxyPrep DNA Gel Extraction Kit (Axygen Biosciences, Union City, CA, United States) strictly adhering to the manufacturer’s instructions. Paired-end Illumina libraries were constructed using genome DNA library preparation technology, followed by sequencing on the Illumina HiSeq2500 system (Thermo Scientific, United States) for the generated libraries. All amplicon sequencing was performed by Shanghai Biozeron Biotechnology Co., Ltd. (Shanghai, China).

### 2.5 Quantitative PCR

The abundance of DAMO-functional genes was analyzed using real-time quantitative PCR (qPCR) technology. The *pmoA* gene and *mcrA* gene were used to quantify the functional gene abundances of nitrite-type and nitrate-type DAMO microbial guilds (Luesken et al., 2011; Vaksmaa et al., 2017). Concurrently, the abundances of bacterial and archaeal 16S rRNA genes were measured to evaluate the relative abundance ratios of DAMO-functional bacteria and archaea within the total bacterial and archaeal communities. SYBR Green dye-based qPCR amplification was conducted on an ABI 7500 Quantitative PCR Instrument (Applied Biosystems, Foster City, CA, United States) using the optimized thermal cycling program. The primer sequences, thermal cycling parameters, and reaction compositions required for qPCR amplification are systematically presented in table form (Supplementary Table 2). Standard curves were established through serial tenfold dilutions of plasmid DNA containing target gene fragments. No-template DNA negative controls were included in identical experimental conditions to exclude exogenous contamination risks. All sample analyses and plasmid validations were performed with three replicate technical runs.

### 2.6 Statistical analysis

Data visualization and statistical analyses were conducted using Origin 2024 and IBM SPSS Statistics 26.0 software platforms, respectively. One-way analysis of variance (ANOVA) was performed to evaluate significant differences in physicochemical properties and functional gene abundances across sediment samples. Microbial community composition differences were visualized through principal coordinate analysis (PCoA) based on Bray–Curtis dissimilarity matrices, implemented in R (version 4.4.0) with the “vegan” package. Spearman’s rank correlation analysis was applied to assess relationships between microbial taxa and environmental variables, with correlation coefficient matrices presented as heatmaps. Microbial network topology parameters were calculated using the “psych” package in R and Gephi software (version 0.10.1). Bacterial and archaeal interaction networks were constructed by including significant correlations where *p* < 0.05 and |R| > 0.6 (Lai et al., 2018). Statistical significance was determined at the threshold of *p* < 0.05 for all analyses.

## 3 Results

### 3.1 Physicochemical characterization of pond sediment

**Figure 1:**
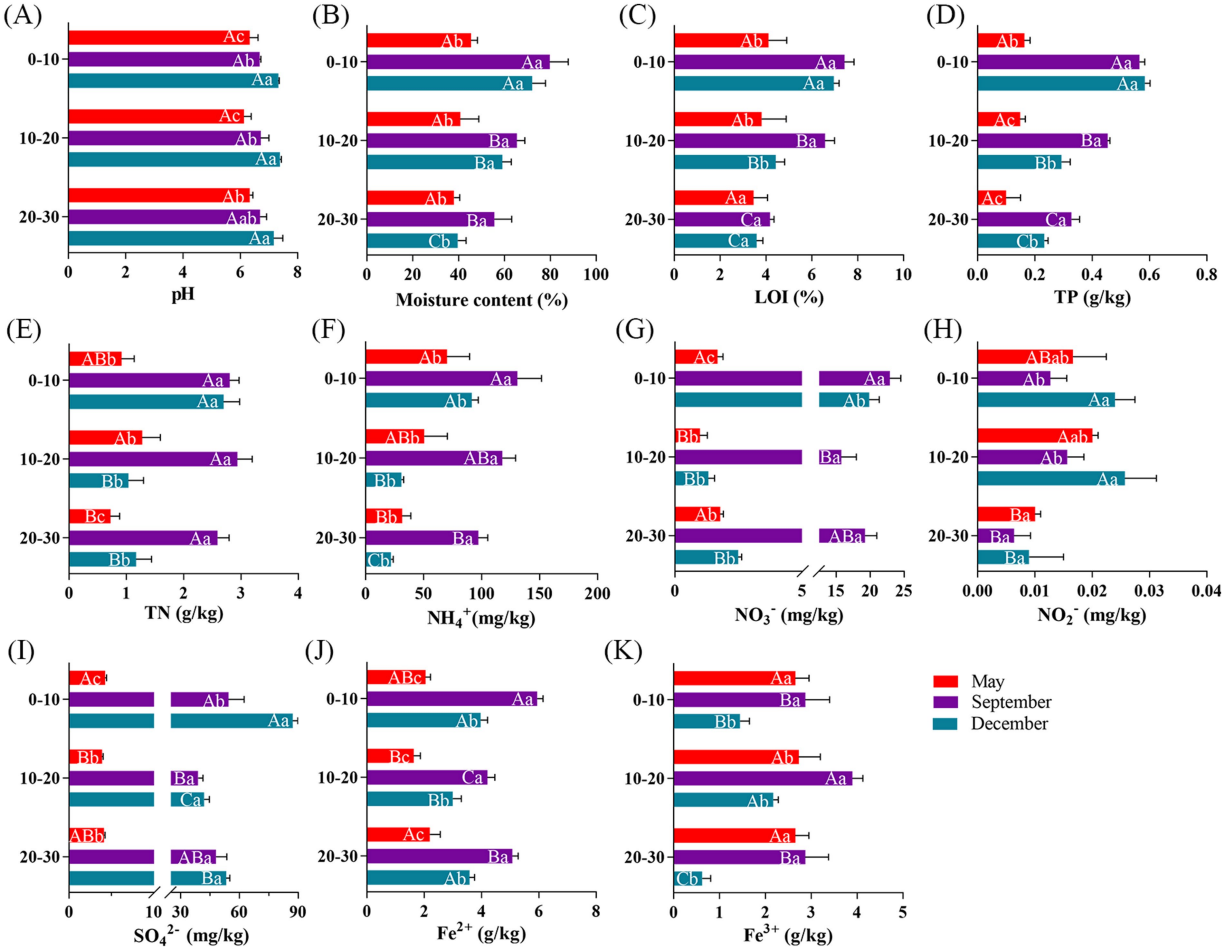
Physical and chemical indicators of sediments under different sampling spatial and temporal conditions. pH **(A)**, moisture content **(B)**, loss on ignition **(C)**, total phosphorus concentration **(D)**, total nitrogen concentration **(E)**, ammoniacal nitrogen concentration **(F)**, nitrate nitrogen concentration **(G)**, nitrite nitrogen concentration **(H)**, sulfate concentration **(I)**, divalent iron ion concentration **(J)**, trivalent iron ion concentration **(K)**. Significant differences (*p* < 0.05) are indicated by the use of different letters on the column graphs. a, b, c represent differences between months; A, B, C represent differences between depths.

The physicochemical properties of the sediments are shown in Figure 1. No significant vertical variation was observed in the sediments’ pH. However, clear seasonal fluctuations were evident, with slightly acidic conditions in May (6.26 ± 0.23) and September (6.69 ± 0.18), shifting to weakly alkaline conditions in December (7.29 ± 0.19). The moisture content, LOI, TP, and NH₄^+^-N concentrations all decreased with increasing depth. In contrast, TN, NO₂^−^-N, and Fe^3+^ concentrations first increased and then decreased with depth, peaking in the middle layers. Conversely, NO₃^−^-N, SO₄^2−^, and Fe^2+^ concentrations showed opposite trends, reaching their lowest levels at 10–20 cm depth. Temporally, most parameters displayed consistently higher values across all sediment depths during September and December compared to May. NO₃^−^-N and NO₂^−^-N showed contrasting distribution patterns both spatially and over time. Along the sediment profile, NO₃^−^-N was lowest at 10–20 cm depth, while NO₂^−^-N peaked in this middle layer. Temporally, NO₃^−^-N levels were significantly higher in September than in May and December at all depths (*p* < 0.05), while NO₂^−^-N showed an opposite seasonal pattern with lower levels in September. SO₄^2−^ displayed distinct spatial and temporal dynamics, reaching minimum levels at 10–20 cm depth but gradually accumulating throughout the monitoring period. Fe^2+^ and Fe^3+^ exhibited opposite vertical distribution patterns, yet both reached their maximum concentrations in September among all sediment layers.

### 3.2 Bacterial and archaeal gene abundance

**Figure 2:**
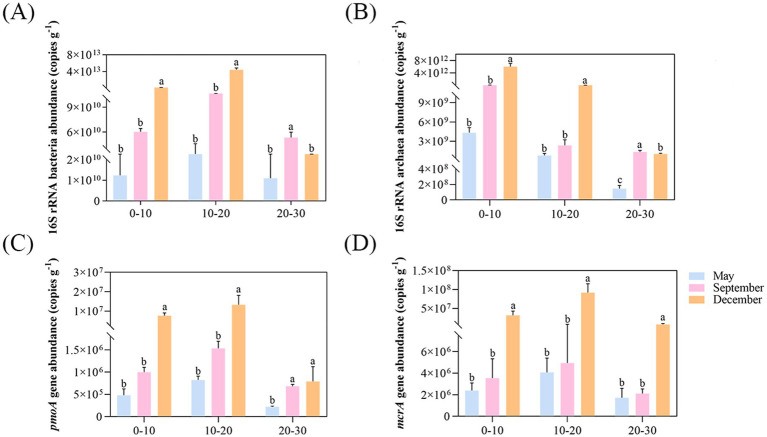
The abundance of microorganisms. The abundance of total bacteria **(A)**, total archaea **(B)**, *pmoA* gene **(C)**, and *mcrA* gene **(D)**. Different letters indicate significant differences (*p* < 0.05).

Quantitative PCR analysis revealed the spatiotemporal distribution characteristics of DAMO bacteria and archaea in pond sediments (Figure 2). The population densities of DAMO bacteria and archaea in pond sediments were relatively low. Throughout the sampling period, bacterial abundance (3.58 × 10^9^–4.72 × 10^13^ copies g^−1^) and archaeal abundance (1.13 × 10^8^–7.12 × 10^12^ copies g^−1^) both exhibited significant spatiotemporal variability. Microbial abundances in surface and middle sediments showed temporal increasing trends, whereas deep-layer samples reached synchronous peaks in September. Vertically, bacterial abundance displayed a distinct depth-dependent bimodal response with a peak at the middle layer, while archaeal abundance demonstrated stable surface aggregation patterns with significantly higher levels compared to deeper layers (Figures 2A,B). Notably, DAMO archaeal abundance (7.15 × 10^5^–1.16 × 10^8^ copies g^−1^) exceeded DAMO bacterial abundance (2.07 × 10^5^–1.89 × 10^7^ copies g^−1^) across all sediment samples, with both groups showing an initial enrichment-later depletion dynamic in vertical profiles, while exhibiting continuous accumulation trends over time (Figures 2C,D).

### 3.3 Diversity and composition of bacterial and archaeal communities

This study employed high-throughput sequencing technology to systematically investigate the diversity and composition of bacterial and archaeal communities in crab pond sediment across different depths and seasons. The alpha diversity indices of sediment bacterial and archaeal communities under different spatio-temporal sampling conditions are presented in Table 1. The Chao1 index was significantly (*p* < 0.05) higher in both bacterial and archaeal communities at the depth of 0–10 cm than those at the depths of 10–20 cm and 20–30 cm in May and December. The Shannon index did not differ significantly between sediment layers throughout the study period, suggesting that while richness varies with depth in some seasons, the relative abundance distribution of dominant taxa remains stable across layers. It is noteworthy that surface layer samples collected in December exhibited significantly higher diversity indices (*p* < 0.05) compared to those from May and September surface samples. This phenomenon may be related to the reduced interspecific competition pressure among microorganisms caused by the drop in water temperature in December.

**Table 1:** The alpha diversity index of bacteria and archaea in different samples.

Time	Depth	Bacteria		Archaea	
		Chao 1	Shannon	Chao 1	Shannon
May	0–10 cm	2991.55 ± 171.23 ^c^	6.55 ± 0.31 ^ab^	3172.14 ± 293.77 ^bc^	5.59 ± 0.76 ^bcd^
May	10–20 cm	2453.66 ± 169.40 ^de^	6.29 ± 0.22 ^ab^	2942.05 ± 103.28 ^bc^	5.53 ± 0.34 ^bcd^
May	20–30 cm	2562.39 ± 131.33 ^d^	6.27 ± 0.37 ^ab^	2852.52 ± 112.43 ^c^	5.42 ± 0.63 ^cd^
September	0–10 cm	2354.96 ± 89.91 ^de^	6.03 ± 0.36 ^bc^	2905.58 ± 46.80 ^bc^	5.22 ± 0.08 ^d^
September	10–20 cm	2457.60 ± 145.22 ^de^	6.01 ± 0.36 ^bc^	3283.39 ± 143.39 ^b^	5.48 ± 0.37 ^bcd^
September	20–30 cm	2242.46 ± 142.50 ^e^	5.51 ± 0.45 ^c^	3128.74 ± 232.64 ^bc^	5.62 ± 0.43 ^bcd^
December	0–10 cm	3817.56 ± 106.40 ^a^	6.81 ± 0.29 ^a^	3927.54 ± 231.87 ^a^	6.65 ± 0.21 ^a^
December	10–20 cm	3396.82 ± 172.68 ^b^	6.74 ± 0.24 ^a^	3747.18 ± 85.38 ^a^	6.37 ± 0.21 ^ab^
December	20–30 cm	2565.48 ± 138.90 ^d^	6.34 ± 0.17 ^ab^	3171.70 ± 120.14 ^bc^	6.27 ± 0.32 ^abc^

Different letters in the same column in the table indicate significant differences (*p* < 0.05).

**Figure 3:**
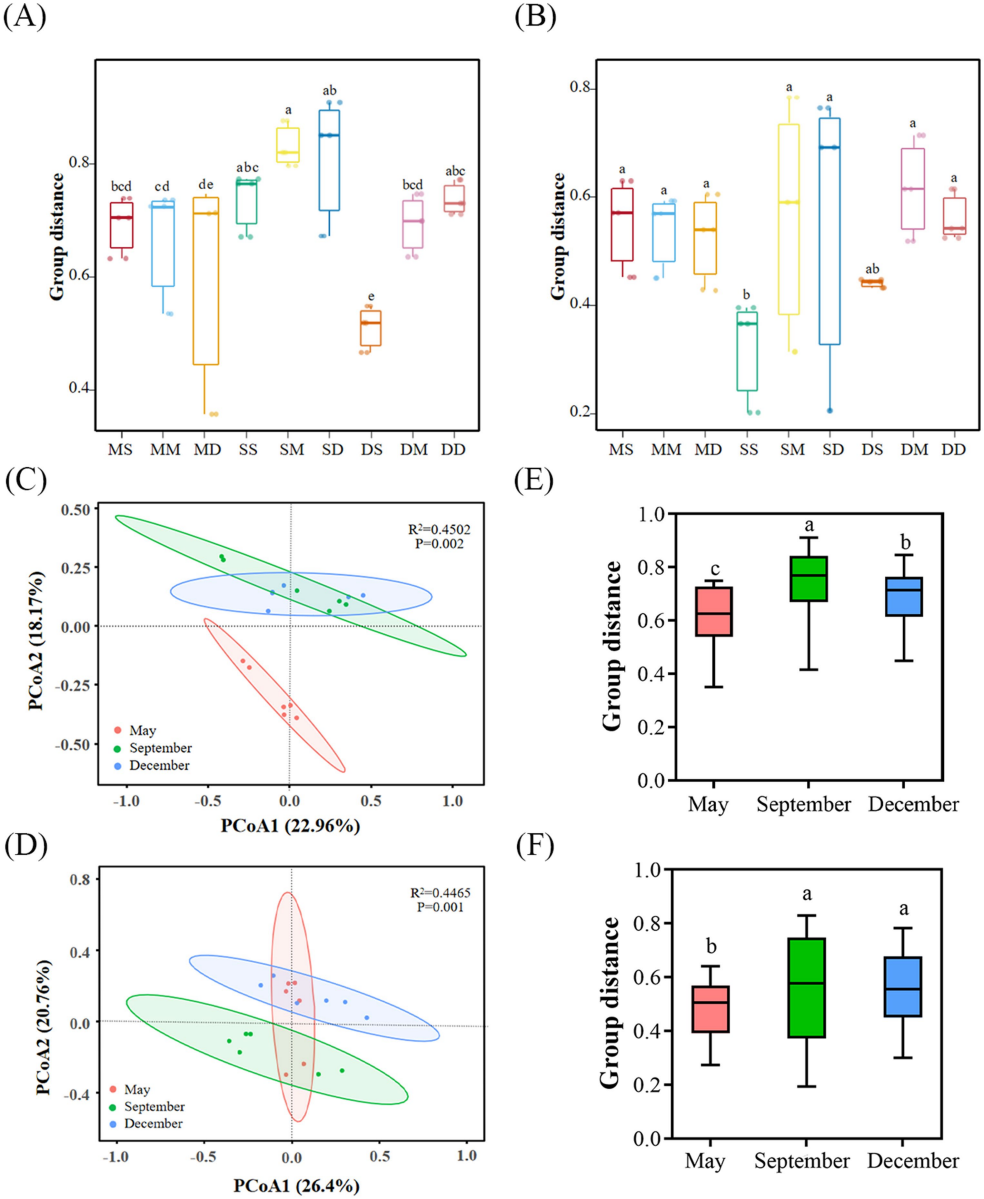
The structure of microbial communities in sediments. **(A,B)**, Comparisons of dissimilarities of bacterial **(A)** and archaeal **(B)** communities across different depths and seasons based on Bray-Curtis distance. **(C,D)**, Principal coordinate analysis (PCoA) of bacterial **(C)** and archaeal **(D)** communities based on Bray-Curtis distance. **(E,F)**, Bray-Curtis distances of bacterial communities **(E)** and archaeal communities **(F)** at different times. Circles in the PCoA plots indicate 95% confidence ellipses. Box plots of the upper and lower quartiles, horizontal lines for the median, whisker lines for the 95% range, and points for outliers are shown. Lowercase letters above the bar graphs indicate significant differences determined by the Kruskal-Wallis H test (*p* < 0.05). MS: May 0–10 cm, MM: May 10–20 cm, MD: May 20–30 cm, SS: September 0–10 cm, SM: September 10–20 cm, SD: September 20–30 cm, DS: December 0–10 cm, DM: December 10–20 cm, DD: December 20–30 cm.

This study reveals the spatial and temporal divergence characteristics of beta diversity of microbial communities through principal coordinate analyses (PCoA). Boxplot data based on Bray-Curtis distances showed a general increasing trend in bacterial beta diversity with sediment depths in September, and that bacterial communities in at different depths in May and September were more similar, whereas bacterial communities at the surface level in December (DS) differed significantly (*p* < 0.05) (Figure 3A). Archaeal communities were more similar at different depths in May and December, and only the surface level in September (SS) differed from the rest of the depths (*p* < 0.05) (Figure 3B). PCoA analyses further elucidated this, bacterial community samples showed a gradient distribution trend on the PCoA1 axis, with May samples concentrated in the negative-axis region, December samples shifted towards the positive axis, while September samples were distributed in the intermediate transition region, and there were significant differences (*p* < 0.05) in the composition of bacterial communities between sampling months, revealing the successive driving effect of seasonal turnover on the beta diversity of bacterial (Figures 3C,E). There were strong similarities between the archaeal communities in September and December, while May samples differed significantly (*p* < 0.05) (Figures 3D,F). Compared to archaeal communities, bacteria were more responsive to seasonal variations.

**Figure 4:**
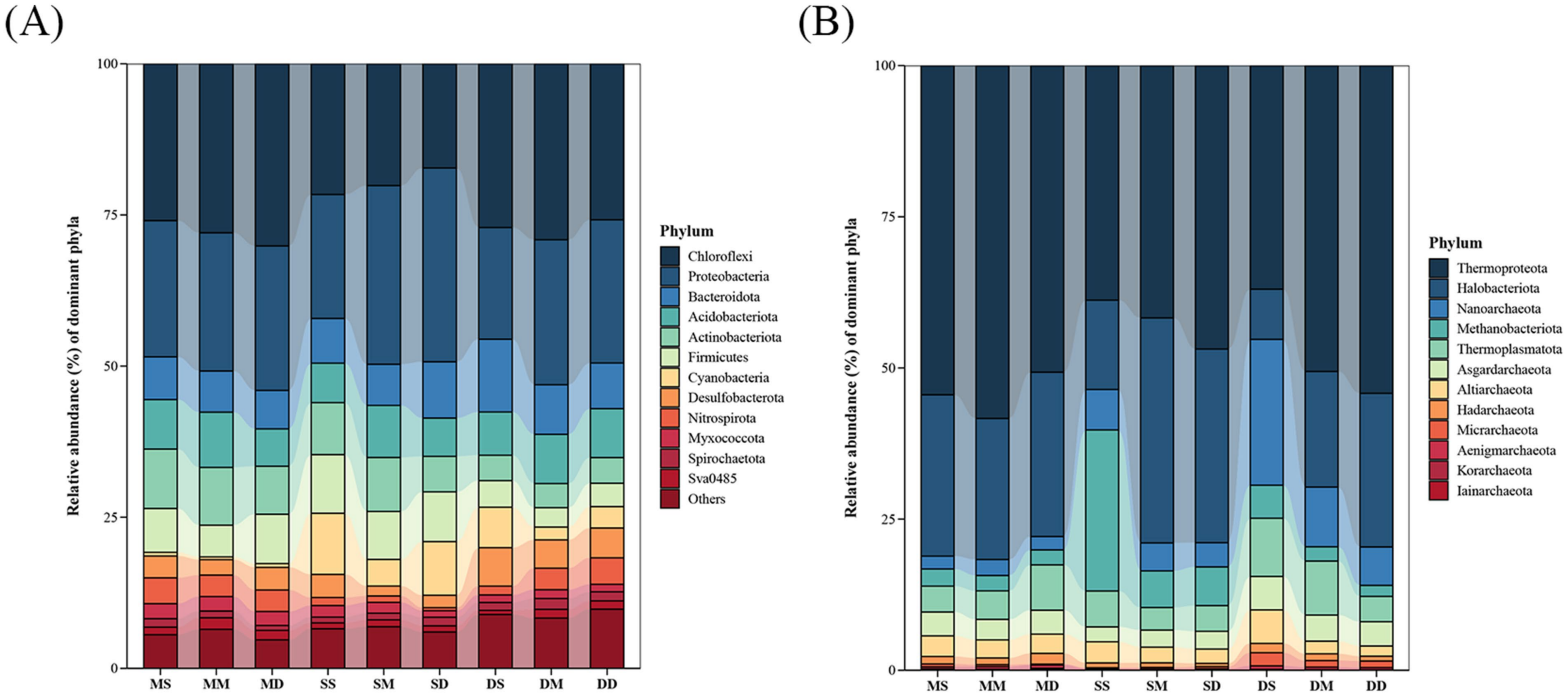
Taxonomic compositions of microbial communities at the phylum level across different seasons and depths in pond sediment. **(A)** Bacterial community. **(B)** Archaeal community. MS: May 0–10 cm, MM: May 10–20 cm, MD: May 20–30 cm, SS: September 0–10 cm, SM: September 10–20 cm, SD: September 20–30 cm, DS: December 0–10 cm, DM: December 10–20 cm, DD: December 20–30 cm.

The taxonomic compositions of bacterial and archaeal communities were shown in Figure 4. A total of 62 bacterial phyla were identified in this study. Among them, 12 bacterial phyla, including Chloroflexi, Proteobacteria, Bacteroidota, Acidobacteriota, Actinobacteriota, Firmicutes, Cyanobacteria, Desulfobacterota, Nitrospirota, Myxococcota, Spirochaetota, and Sva0485, accounted for more than 90% of the total sequences (Figure 4A). The relative abundances of the dominant bacterial phyla exhibited temporal heterogeneity. In May, there was an obvious enrichment of Chloroflexi, Acidobacteriota, Nitrospirota, Actinobacteriota, and Myxococcota in sediment across all depths. Chloroflexi, primarily functioning as anaerobic heterotrophs, decomposed complex and recalcitrant organic matter, and dominated the input of organic matter in sediments when feeding intensity was low in May. Nitrospirota included nitrite-oxidizing bacteria, which maintained nitrogen balance in the early pond ecosystem through nitrification. In September, Proteobacteria, Firmicutes, and Cyanobacteria reached their annual peaks in sediment across all depths. Cyanobacteria thrived on excess nutrients, and their proliferation reflected a slight eutrophication. In December, Bacteroidota and Desulfobacterota showed obvious enrichment in sediment across all depths. Bacteroidota specialized in decomposing high-molecular-weight organic matter such as crab shells. Desulfobacterota regulated anaerobic carbon and sulfur cycles, which could both inhibit organic matter accumulation and alleviate hydrogen sulfide toxicity. A total of 12 archaeal phyla were identified in this study. These included Thermoproteota, Halobacteriota, Nanoarchaeota, Methanobacteriota, Thermoplasmatota, Asgardarchaeota, Altiarchaeota, Hadarchaeota, Micrarchaeota, Aenigmarchaeota, Korarchaeota, and Iainarchaeota (Figure 4B). The relative abundances of the dominant archaeal phyla were characterized by seasonal vertical stratification. In September and December, the relative abundances of Halobacteriota and Thermoproteota increased with sediment depth, whereas Altiarchaeota, Methanobacteriota, and Nanoarchaeota exhibited an opposite trend. Methanobacteriota belonged to methanogenic archaea and required strictly anaerobic environments. Therefore, their relatively high abundance in the surface layer in September and December might be related to the increased organic input and temporary anoxia caused by high decomposition rates. Notably, in May, Thermoproteota reached its peak relative abundance at 10–20 cm depth, while Altiarchaeota and Halobacteriota showed their lowest relative abundances at this layer.

**Figure 5:**
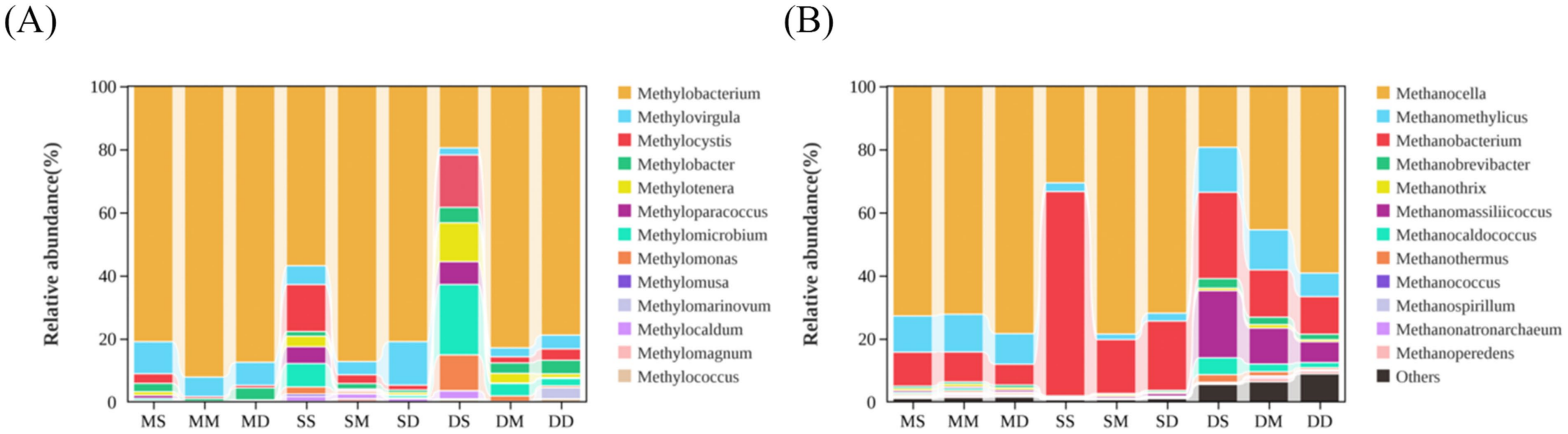
Relative abundance of methane-metabolizing microorganisms at the genus level. **(A)** Methane-metabolizing bacterial communities. **(B)** Methane-metabolizing archaeal communities. MS: May 0–10 cm, MM: May 10–20 cm, MD: May 20–30 cm, SS: September 0–10 cm, SM: September 10–20 cm, SD: September 20–30 cm, DS: December 0–10 cm, DM: December 10–20 cm, DD: December 20–30 cm.

In addition, particular attention was paid to the total relative abundance of all methane-metabolizing microorganisms at the genus level (Supplementary Figure 2). For all identified methane-metabolizing microorganisms at the genus level, their relative abundances were calculated and statistically analyzed, defined as the abundance of each microorganism divided by the total abundance of all methane-metabolizing microorganisms at the genus level. It was found that there were more species of methane-metabolizing archaea than bacteria in all samples (Figure 5). Among methane-metabolizing bacterial communities, *Methylobacterium*, *Methylovirgula*, *Methylocystis*, and *Methylobacter* exhibited higher relative abundances compared to other taxa. Comparative analysis of relative abundances across seasons and sediment depths revealed higher proportions of *Methylotenera*, *Methyloparacoccus*, *Methylomicrobium*, and *Methylomonas* in surface sediment samples collected during September and December compared to May surface samples (Figure 5A). In the archaeal domain, *Methanocella*, *Methanomethylicus*, and *Methanobacterium* were dominant across all samples. *Methanobrevibacter*, *Methanomassiliicoccus*, and *Methanocaldococcus* had higher relative abundances across all depth samples in December compared to the other 2 months (Figure 5B).

### 3.4 Microbial cross-domain ecological network characteristics

**Figure 6:**
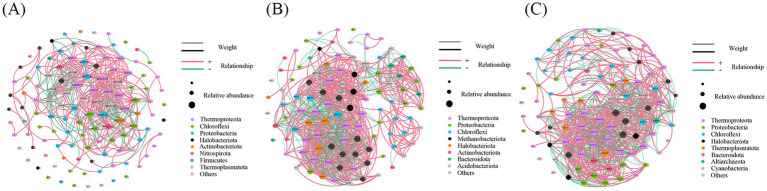
Interaction network diagram of bacteria-archaea in sediments. **(A)** May, **(B)** September, **(C)** December.

A cross-domain ecological network between bacteria and archaea was constructed based on SparCC correlations of OTUs to explore potential microbial interactions. The three constructed networks exhibited distinct structural and topological characteristics (Figure 6). It was found that the majority of nodes in these three networks belonged to four dominant phyla (node proportion ≥ 5%). Among them, Thermoproteota, Chloroflexi, and Proteobacteria were the predominant phyla in the May network, while Nitrospirota and Firmicutes showed higher proportions compared to the other two networks. In the September network, Thermoproteota, Proteobacteria, and Chloroflexi dominated, whereas Methanobacteriota and Acidobacteriota exhibited higher proportions than in the other networks. The December network was primarily dominated by Thermoproteota, Proteobacteria, and Chloroflexi, with Cyanobacteria and Altiarchaeota displaying elevated proportions relative to the other two networks. These results reveal distinct compositional and structural differences among the three cross-domain bacteria-archaea networks.

In this study, three networks were constructed using identical thresholds. The results demonstrated that the cross-domain bacteria-archaea networks for May, September, and December contained 120 nodes with 535 edges (average degree of 8.92), 105 nodes with 950 edges (average degree of 18.10), and 102 nodes with 903 edges (average degree of 17.71), respectively (Table 2). The proportion of positive correlations exhibited a progressive decline over time, while negative correlations showed a corresponding increase, indicating enhanced competitive interactions among microbial taxa in September and December. A concurrent rise in modularity and fall in average path length suggested improved network efficiency and more compact structural organization in December. Although network density improved, the average weighted degree declined from 7.05 to 4.52, demonstrating weakened individual connection strength despite enhanced overall connectivity during December. Notably, the observed reduction in average clustering coefficient in December may reflect topological reorganization of node connectivity patterns.

**Table 2:** Topological parameters of microbial community co-occurrence networks.

Topological parameters	May	September	December
Number of nodes	120	105	102
Number of edges	535	950	903
Number of positively correlated edges	411 (76.82%)	596 (62.74%)	539 (59.69%)
Number of negatively correlated edges	124 (23.18%)	354 (37.26%)	364 (40.31%)
Average degree	8.92	18.10	17.71
Average weighted degree	7.05	6.97	4.52
Diameter	9	11	7
Density	0.08	0.17	0.18
Modularity	0.91	2.86	4.84
Average clustering coefficient	0.70	0.75	0.68
Average path length	3.13	3.23	2.58

### 3.5 Correlations between microorganisms and environmental factors

**Figure 7:**
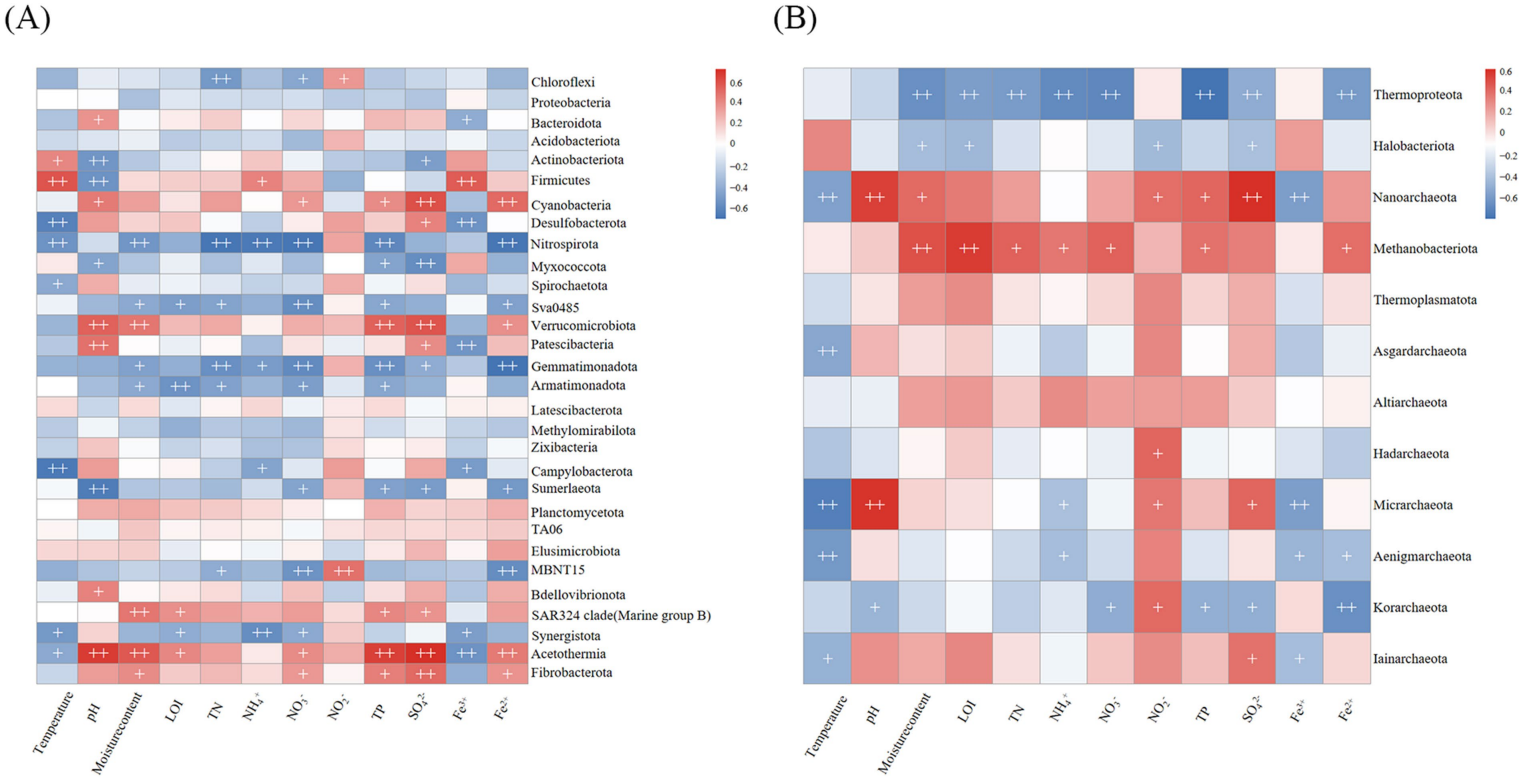
Spearman correlations of environmental factors with relative abundances of the dominant phyla. **(A)** Bacterial communities. **(B)** Archaeal communities. + *p* < 0.05, ++ *p* < 0.01.

Spearman correlation analyses were employed to detect the key environmental factors influencing the compositions of bacterial and archaeal communities in pond sediments (Figure 7). The results indicate that pH, nitrate nitrogen, total phosphorus, sulfate, and iron influence a broader range of bacterial phyla (Figure 7A). pH showed significant positive or negative correlations with multiple bacterial phyla, including Bacteroidota, Actinobacteriota, and Firmicutes, confirming the pH-mediated niche differentiation theory. Nitrogen cycle-related factors, including total nitrogen, ammonium nitrogen, and nitrate nitrogen, exhibited consistent and significant negative correlations with the relative abundances of Nitrospirota and Gemmatimonadota. Sulfate concentration demonstrated a statistically significant positive correlation with the relative abundance of Desulfobacterota. Notably, Nitrospirota and Sva0485 exhibited consistent response patterns to environmental factors, whereas Cyanobacteria demonstrated opposing correlation trends.

For archaeal communities, temperature, nitrite nitrogen, and sulfate exerted more pronounced influences on a greater number of phyla (Figure 7B). Temperature exhibited a significant negative correlation with the relative abundances of the majority of archaeal phyla. Total nitrogen concentration demonstrated significant effects on the relative abundances of only Thermoproteota and Methanobacteriota. Thermoproteota and Methanobacteriota showed significantly inverse correlation patterns in response to environmental factors, suggesting potential niche differentiation or interactive mechanisms. Multiple environmental parameters, including moisture content, loss on ignition, total nitrogen, ammonium nitrogen, nitrate nitrogen, total phosphorus, and divalent iron ion, showed significant positive correlations with the relative abundance of Methanobacteriota.

**Figure 8:**
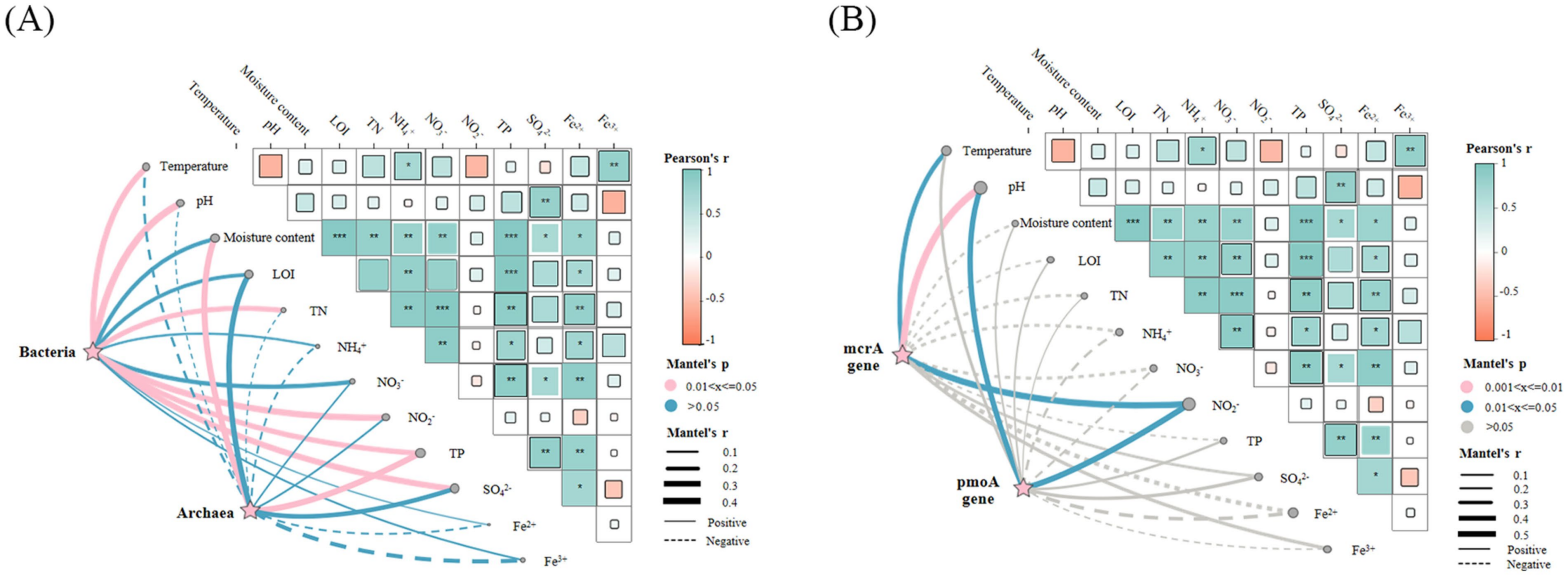
Heat map of the mantel test correlation between microbial abundance and environmental factors. **(A)** 16S rRNA bacteria and archaea. **(B)** DAMO bacterial *pmoA* gene and DAMO archaeal *mcrA* gene. * *p* < 0.05, ** *p* < 0.01, *** *p* < 0.001.

Herein, Mantel tests were employed to reveal significant associations between environmental variables and the abundances of bacterial 16S rRNA, archaeal 16S rRNA, *pmoA*, and *mcrA* genes (Figure 8). The analysis demonstrated differential responses of these gene abundances to environmental factors. The bacterial 16S rRNA gene abundance showed significant positive correlations with temperature, pH, total nitrogen, nitrite nitrogen, total phosphorus, and sulfate, as revealed by Mantel tests. In contrast, archaeal 16S rRNA gene abundance exhibited distinct environmental responsiveness, demonstrating significant positive associations specifically with moisture content and total phosphorus. The *pmoA* gene abundance exhibited a distinct environmental response pattern, characterized by significant positive correlations with pH and nitrite nitrogen, alongside a significant negative correlation with iron ions. Concurrently, *mcrA* gene abundance showed significant positive correlations with temperature, pH, and nitrite nitrogen, but negative correlations with total nitrogen and total phosphorus.

## 4 Discussion

### 4.1 Differentiation characteristics of DAMO microbial abundance

The qPCR results demonstrated that DAMO microorganisms exhibited widespread distribution in crab aquaculture pond sediments, with both bacterial and archaeal lineages catalyzing anaerobic methane oxidation detected at all sampling sites. The *pmoA* gene abundance of DAMO bacteria ranged from 2.07 × 10^5^ to 1.89 × 10^7^ copies g^−1^, which were comparable to those observed in riverine sediments (9.3 × 10^5^–1.5 × 10^7^ copies g^−1^), mangrove wetland sediments (2.07 × 10^6^–3.38 × 10^7^ copies g^−1^), and riparian zone sediments (3.06 × 10^6^–1.22 × 10^7^ copies g^−1^), but higher than those measured in marine sediments (10^4^–10^5^ copies g^−1^) (Zhang et al., 2018; Shen et al., 2019; Cheng et al., 2022; Hu et al., 2025). The *mcrA* gene abundance of DAMO archaea displayed a broader range (7.15 × 10^5^–1.16 × 10^8^ copies g^−1^), exceeding those reported in marine sediments (2.5 × 10^4^ copies g^−1^) and estuarine intertidal zones (4.3 × 10^4^–1.2 × 10^5^ copies g^−1^) (Vaksmaa et al., 2017; Wang J. et al., 2019). These findings suggest that freshwater ecosystems, particularly eutrophic pond systems, may provide more favorable ecological niches for DAMO microorganisms compared to marine environments, where substrate availability and microenvironmental characteristics likely constitute critical selective pressures.

DAMO bacteria and archaea coexisted in crab aquaculture ponds, potentially serving as synergistic partners in executing the DAMO process. Both DAMO bacteria and archaea exhibited identical temporal distribution patterns, with their abundances gradually increasing throughout the study period. This seasonal trend aligns with the distribution patterns of DAMO functional microorganisms observed in urban sludge, where abundance peaks during autumn and winter but declines in spring and summer, suggesting that temperature and nutrient concentrations may influence their metabolic activities (Xu et al., 2017; Xu et al., 2020). Spatially, both the *pmoA* gene abundance of DAMO bacteria and the *mcrA* gene abundance of archaea reached their peak levels in the middle sediment layer (10–20 cm depth). The abundance of the *pmoA* gene exhibited significant differences across all sampled depths during May and September, whereas in December, significant variations were only observed between the bottom layer (20–30 cm) and other depths. Differently, the abundance of the *mcrA* gene in May was significantly different at depths of 10–20 cm and 20–30 cm, whereas no significant variations were observed between depths in September, and the abundance of the *mcrA* gene in December was significantly different from the other depths only at depths of 10–20 cm. Previous studies have demonstrated that animal perturbations influence methane emissions in aquatic systems, implying that the activity of *Eriocheir sinensis* may affect methane flux in pond ecosystems (Oliveira Junior et al., 2019; Colina et al., 2021; Dong et al., 2023). As methane serves as the primary substrate for DAMO processes, elevated methane concentrations could potentially reshape the spatial distribution of DAMO functional microbial communities (Ding et al., 2016). The foraging and burrowing behaviors of crabs could disrupt sediment structure, which can increase sediment porosity and oxygen penetration. The increased porosity enhances oxygen flux across the sediment–water interface, while dissolved oxygen exerts strong inhibitory effects on DAMO microbial activity (Lou et al., 2020). Consequently, the abundance of DAMO functional microorganisms in surface sediments shows a significant reduction compared to that in the middle sediment layers. DAMO archaea, compared to bacteria, exhibit stricter anaerobic niche preferences, resulting in no difference in abundance of DAMO archaea between 0–10 cm and 10–20 cm during the disturbance period (May and September) (Ding and Zeng, 2021). By December, crab production is over, and resuspension of sediment due to crab activity is reduced. The middle layer sediment, characterized by prolonged anaerobic conditions, develops a stable denitrification microenvironment with persistent redox conditions and slow organic carbon release. This microenvironment provides a selective growth advantage for DAMO archaea, whereas DAMO bacteria exhibited comparable abundances in the sediment of the surface and middle layers.

### 4.2 Dynamics and diversity differences of DAMO microbiota

Microbial communities are core components of aquaculture ecosystems, where their diversity and functional activity directly influence the health of cultured organisms, environmental stability, and biogeochemical element cycling. High-throughput sequencing technology enables comprehensive coverage of microbial diversity (Shen et al., 2017). In this study, microbial diversity and composition across all collected sediment samples were analyzed via high-throughput sequencing. The results demonstrated that bacterial diversity in crab pond sediments exhibited seasonal variations, whereas its vertical distribution remained relatively stable. Specifically, the depth gradient of microbial diversity observed in May and December may stem from the fact that the surface sediment layer receives more organic inputs and maintains higher dissolved oxygen levels, which provide richer carbon sources and a suitable aerobic environment for heterotrophic and aerobic microorganisms, thereby supporting a more diverse community (Keet et al., 2019). The highest Chao1 diversity indices were recorded in December. The peak Chao1 index in December is likely associated with the cumulative accumulation of nutrients throughout the aquaculture cycle. By December, the contents of organic carbon, total nitrogen, and other nutrients in the sediment increase significantly, offering more abundant substrates for the growth and reproduction of various microorganisms. In contrast, the absence of depth-related differences in diversity in September may reflect the homogenization of sediment environmental conditions. High summer temperatures not only accelerate microbial metabolic activity but also promote the diffusion of nutrients across different depths, eliminating resource limitations that would otherwise drive the vertical stratification of microbial communities (Orwin et al., 2016). Previous studies have identified sediment moisture content as a critical regulator of bacterial communities, with higher moisture levels favoring bacterial diversity (Nakamura et al., 2003; Banerjee et al., 2016; Keet et al., 2019). Substrates in sediments similarly affect bacterial abundance and diversity (Orwin et al., 2016; Santonja et al., 2017). In the studied crab pond sediments, organic matter decomposition by heterotrophic bacteria provided methane precursors for methanogens, which in turn produced methane, forming a substrate chain that supported DAMO survival.

Compared to bacterial communities, archaeal diversity exhibited a delayed response to seasonal variations, with significant differences observed only in May relative to other months. These differences in responsiveness were likely closely related to metabolic traits. Bacteria typically had faster growth rates and a broader range of substrate utilization capabilities, allowing them to adapt quickly to seasonal changes in temperature and nutrients. In contrast, archaea often had slower metabolic rates and stricter requirements for environmental conditions, which resulted in a delayed response to seasonal fluctuations (Endara and Coley, 2011). Vertical distributions showed no significant gradient in archaeal diversity with depth, contrasting sharply with classical patterns reported in previous studies where microbial diversity typically displayed positive or negative correlations with sediment depth (Gong et al., 2022; Zhu et al., 2023). This stability may arise from the homogeneity of environmental gradients and absence of resource limitations within the sediment vertical profile, which collectively provide stable and favorable conditions for microbial survival (Coley et al., 1985; Endara and Coley, 2011). Community composition analysis identified Thermoproteota, Halobacteriota, Nanoarchaeota, and Methanobacteriota as dominant phyla in the sediments. While inter-sample archaeal community compositions showed high similarity, relative abundances of specific taxa varied considerably between samples. Notably, Methanobacteriota abundance increased markedly across all sediment layers in September, when nutrients and temperature were at higher levels, suggesting that nutrient enrichment and optimal temperature synergistically enhance its relative abundance. The relative abundances of *Methanocella*, *Methanomethylicus*, and *Methanobacterium* at the forefront of methane-metabolizing archaea, consistent with previous findings of *Methanocella*’s high abundance in pond sediments (Silveira et al., 2021). The methanotrophic archaeon *Methanoperedens*, implicated in methane oxidation, exhibited increased abundance in December, implying that methane metabolism can still take place at low temperature (Wallenius et al., 2021). However, significant knowledge gaps remain in the composition and functional characterization of archaeal communities in crab aquaculture systems, necessitating integrated meta-transcriptomic and *in situ* metabolic flux analyses to elucidate their adaptive mechanisms under aquacultural stressors.

Community composition analysis revealed that Proteobacteria, Chloroflexi, and Bacteroidota dominated sediment microbial communities, collectively accounting for over 50% relative abundance, consistent with findings from comparable crab pond studies (Gao et al., 2023). Notably, Cyanobacteria abundance increased significantly in September and December compared to that in May, suggesting intensified eutrophication during these periods. Previous studies have identified some genera of proteobacteria (e.g., *Methylcystis*) that carry the *mmoX* gene encoding the *α*-subunit of the soluble methane monooxygenase hydroxylase complex, indicating efficient methane oxidation capacity (Jhala et al., 2014; Nho et al., 2018). *Methylobacterium* exhibited the highest relative abundance among these methanotrophic bacteria. The variation in relative abundance of methanotrophic bacteria could be jointly regulated by substrate availability and interspecific competition relationships. The elevated *Methylocystis* abundance in September and December may correlate with nitrogen accumulation, as some *Methylocystis* members possess diazotrophic capabilities, aligning with higher nitrogen content observed during these months (Dam et al., 2013; Collavino et al., 2014). Additionally, *Methylocystis* may gain a competitive advantage through chitin utilization as a nitrogen source (Fan et al., 2019).

Microbial co-occurrence networks clearly revealed interactions among microbial taxa at the phylum level. As the core dominant phylum across the three networks, Proteobacteria included aerobic methanotrophic bacteria, which competed with DAMO bacteria for methane resources, and notably, the average degree of the September and December networks was significantly higher than that of the May network, a topological characteristic indicating that Proteobacteria interacted more frequently with other taxa in these two months, likely including competition with DAMO bacteria for methane (Zhang et al., 2021). In contrast, May-enriched Chloroflexi were anaerobic heterotrophs that decomposed complex recalcitrant organic matter in sediments into small-molecule substrates, and their higher proportion of positive correlations with other taxa in the May network suggested this synergistic relationship indirectly provided sufficient methane substrates for DAMO archaea by increasing methane precursor availability, supporting early-season DAMO survival and proliferation (Zhang et al., 2019). Nitrospirota converted nitrite to nitrate. Weak negative correlations in May enabled DAMO to utilize nitrite efficiently, but intensified competition in September reduced nitrite availability, limiting DAMO microbial growth (Umezawa et al., 2020). Additionally, Desulfobacterales, typical sulfate-reducing bacteria, competed with DAMO archaea for sulfate as an alternative electron acceptor, further impairing their metabolic efficiency (Wang et al., 2023). Cyanobacteria, more abundant in December, produced oxygen in surface sediments via photosynthesis, disrupting the strictly anaerobic environment required by DAMO archaea and inhibiting their surface survival and activity (Stebegg et al., 2023). This is consistent with the results of our study that the abundance of DAMO archaea peaked in the mid-layers in December.

### 4.3 Effects of environmental factors on the abundance of DAMO microorganisms

A significant positive correlation between DAMO bacterial abundance and pH was observed in the sediment of *Eriocheir sinensis* ponds. In our study, the highest pH was 7.4, corresponding to the highest abundance, which is consistent with the reported optimal pH of 7.6 (Zhang et al., 2018; Li et al., 2020). This similarity highlighted the conservative physiological adaptation of DAMO bacteria to pH across different aquatic environments. Specifically, the pH range of 7.0–7.8 maintained both the structural stability and catalytic activity of key functional enzymes in DAMO bacteria. From a physiological mechanism perspective, neutral to slightly alkaline conditions prevented protonation of the enzymes’ active centers and denaturation of functional proteins, which in turn ensured the efficiency of methane oxidation and nitrogen reduction processes (Xu Y. et al., 2018). As a core electron acceptor for DAMO bacteria, NO₂^−^ concentration showed a significant positive correlation with *pmoA* gene abundance, corroborating its central role in methane oxidation. This result corroborated the well-documented central role of NO₂^−^ in the DAMO bacterial metabolic pathway, which has been consistently observed in natural ecosystems. The consistency here reflected the universality of NO₂^−^ limitation for DAMO bacterial proliferation, sufficient NO₂^−^ availability was a prerequisite for their growth. Notably, a negative correlation between DAMO bacterial abundance and NH₄^+^ was detected, contradicting previous reports of positive associations (Shen et al., 2014; Long et al., 2017). This discrepancy could be attributed to the unique environmental context of high-intensity crab aquaculture ponds, which differed fundamentally from the study systems in prior work. Crab ponds in this study exhibited substantially higher NH₄^+^ levels attributable to intensive feeding regimes and crab excrement accumulation, fostering intense niche competition between DAMO bacteria and ammonia-oxidizing microorganisms (AOMs). AOMs possessed a greater affinity for NH₄^+^ than DAMO bacteria, enabling them to outcompete DAMO bacteria for NH₄^+^ resources (Chen et al., 2021). Furthermore, aerobic methanotrophs may indirectly influence the dynamics of the DAMO bacterial community through substrate competition, further suppressing DAMO bacterial proliferation when NH₄^+^ was limited (Kits et al., 2015). This competitive interplay that was unique to high-intensity aquaculture systems explained the negative correlation observed here, while distinguishing our findings from those in low-nutrient, low-competition environments.

For DAMO archaea, the abundance of the *mcrA* gene showed a significant positive correlation with temperature, indicating greater sensitivity to temperature fluctuations than DAMO bacteria. From an ecological and physiological perspective, elevated temperature enhanced DAMO archaeal growth and proliferation through two key mechanisms. First, it increased the activity of methane-oxidizing enzymes, whose catalytic efficiency doubled with every 10 °C increase within the optimal range; second, it accelerated cell membrane fluidity and nutrient transport rates, which promoted the uptake of methane and electron acceptors (Hu et al., 2009). The positive correlation between pH and *mcrA* gene abundance aligns with physiological evidence. DAMO archaea demonstrate optimal growth in enrichment cultures at pH 7.0–7.5. Unlike DAMO bacteria, however, previous studies have noted that DAMO archaea can maintain metabolic activity under highly alkaline conditions (pH > 8.0), likely due to their ability to synthesize alkaline shock proteins that protect functional enzymes (Fierer and Jackson, 2006). In the crab ponds of this study, pH rarely exceeded 7.8, so this alkaline tolerance was not observed. However, the consistency in optimal pH further confirmed the shared pH adaptation of DAMO archaea across different habitats. Although NO₃^−^ is an electron acceptor for DAMO archaea during methane oxidation, no significant association was observed between NO₃^−^ concentration and *mcrA* abundance in this study. This differed from the reported positive correlation in lacustrine sediments (Weber et al., 2017). This difference was likely driven by two factors. First, the metabolic flexibility of DAMO archaea in crab pond sediments has been shown: DAMO archaea could utilize alternative electron acceptors (such as Fe^3+^, SO₄^2−^) when NO₃^−^ was limited or fluctuating (Ettwig et al., 2016; Cai et al., 2018; Nie et al., 2021). In this study, sediment Fe^3+^ concentration was relatively high, providing a substitute electron acceptor that buffered the impact of NO₃^−^ fluctuations. Second, crab ponds had highly variable NO₃^−^ concentrations across seasons, which may have masked the correlation between NO₃^−^ and mcrA abundance (Wang et al., 2017).

In summary, this study explored the distribution characteristics of DAMO microorganisms in *Eriocheir sinensis* pond sediments across temporal and vertical scales, revealing the driving effects of pH, temperature, nitrogen, and other environmental factors, providing basic data for research on DAMO microorganisms in this habitat. However, the study is limited to internal analysis of a single type of crab pond, lacking horizontal comparison between different ponds, which restricts the general applicability of the conclusions. Future research could expand the horizontal scope to clarify the universality and specificity of their distribution, and strengthen the linkage analysis of aquaculture activities, methane flux, and DAMO microorganisms, providing more precise basis for ecological aquaculture strategies to reduce methane emissions and purify nitrogen in ponds by regulating DAMO.

## 5 Conclusion

This study employed molecular biological techniques and high-throughput sequencing platforms to confirm the existence of DAMO processes in crab aquaculture pond sediments. The results demonstrated that DAMO bacteria and archaea form a stable coexisting system in sediments, exhibiting significant spatiotemporal heterogeneity in abundance, with archaeal abundance consistently exceeding bacterial abundance. The abundance of DAMO bacteria and archaea was shaped by multivariate factors, with temperature, pH, and NO_2_^−^ concentrations identified as critical regulators of their functional microbial abundance and spatial distribution. Furthermore, microbial community composition and diversity displayed pronounced spatiotemporal differentiation, with seasonal variations exerting a stronger influence than vertical gradients. Methane-metabolizing archaea exhibited greater diversity compared to methane-metabolizing bacteria. Notably, microbial communities displayed elevated diversity and more complex interspecies interactions throughout the later stages of the aquaculture cycle. Collectively, this study elucidates the spatiotemporal distribution patterns of DAMO microorganisms in crab pond sediments, providing theoretical strategies for mitigating greenhouse gas emissions from aquatic systems.

## Data Availability

The raw reads were submitted to the NCBI Sequence Read Archive (SRA) at https://www.ncbi.nlm.nih.gov/sra under accession numbers PRJNA1274850 for bacteria and PRJNA1275853 for archaea, as detailed in Supplementary Table 1.
