# Supplementary material for: Spatiotemporal variations and environmental drivers of denitrifying anaerobic methane oxidizers in Eriocheir sinensis pond sediments
Source: Front Microbiol. 2025 Sep 25;16:1679266. doi: 10.3389/fmicb.2025.1679266 (PMC12509068; doi:10.3389/fmicb.2025.1679266)
Supplement: Supplementary file 1 [file Supplementary_file_1.docx]

**Spatiotemporal variations and environmental drivers of denitrifying anaerobic methane oxidizers in *Eriocheir sinensis* pond sediments**

Hongfei Zhang ^1,2^, Huimin Xu ^2,*^, Honghai Zou ^3^, Limin Fan ^1,2^, Xiangke Fan ^3^, Dandan Li ^2^, Longxiang Fang ^2^, Zhuping Liu ^2^, Hao Zheng ^3^, Liping Qiu ^2^, Shunlong Meng ^1,2,*^

1. Wuxi Fishery College, Nanjing Agricultural University, Wuxi 214081, China.

2. Freshwater Fisheries Research Center, Chinese Academy of Fishery Sciences; Scientific Observing and Experimental Station of Fishery Resources and Environment in the Lower Reaches of the Changjiang River, Wuxi 214081, China.

3. Jiangsu Fisheries Technology Extension Center, Nanjing 210036, China.

* Correspondence authors:
Huimin Xu, E-mail: xuhuimin@ffrc.cn
Shunlong Meng, E-mail: mengsl@ffrc.cn

**Supplementary Table 1. Summary information of the data set and the sample accession ID**

| **Sample name** | **BioProject** | **Accession** | **Sample type** |
| --- | --- | --- | --- |
| a13 | PRJNA1274850 | SAMN48996429 | Crab aquaculture pond sediment |
| a14 | PRJNA1274850 | SAMN48996430 | Crab aquaculture pond sediment |
| a15 | PRJNA1274850 | SAMN48996431 | Crab aquaculture pond sediment |
| a16 | PRJNA1274850 | SAMN48996432 | Crab aquaculture pond sediment |
| a17 | PRJNA1274850 | SAMN48996433 | Crab aquaculture pond sediment |
| a18 | PRJNA1274850 | SAMN48996434 | Crab aquaculture pond sediment |
| a19 | PRJNA1274850 | SAMN48996435 | Crab aquaculture pond sediment |
| a20 | PRJNA1274850 | SAMN48996436 | Crab aquaculture pond sediment |
| a21 | PRJNA1274850 | SAMN48996437 | Crab aquaculture pond sediment |
| b13 | PRJNA1274850 | SAMN48996456 | Crab aquaculture pond sediment |
| b14 | PRJNA1274850 | SAMN48996457 | Crab aquaculture pond sediment |
| b15 | PRJNA1274850 | SAMN48996458 | Crab aquaculture pond sediment |
| b16 | PRJNA1274850 | SAMN48996459 | Crab aquaculture pond sediment |
| b17 | PRJNA1274850 | SAMN48996460 | Crab aquaculture pond sediment |
| b18 | PRJNA1274850 | SAMN48996461 | Crab aquaculture pond sediment |
| b19 | PRJNA1274850 | SAMN48996462 | Crab aquaculture pond sediment |
| b20 | PRJNA1274850 | SAMN48996463 | Crab aquaculture pond sediment |
| b21 | PRJNA1274850 | SAMN48996464 | Crab aquaculture pond sediment |
| c13 | PRJNA1274850 | SAMN48996483 | Crab aquaculture pond sediment |
| c14 | PRJNA1274850 | SAMN48996484 | Crab aquaculture pond sediment |
| c15 | PRJNA1274850 | SAMN48996485 | Crab aquaculture pond sediment |
| c16 | PRJNA1274850 | SAMN48996486 | Crab aquaculture pond sediment |
| c17 | PRJNA1274850 | SAMN48996487 | Crab aquaculture pond sediment |
| c18 | PRJNA1274850 | SAMN48996488 | Crab aquaculture pond sediment |
| c19 | PRJNA1274850 | SAMN48996489 | Crab aquaculture pond sediment |
| c20 | PRJNA1274850 | SAMN48996490 | Crab aquaculture pond sediment |
| c21 | PRJNA1274850 | SAMN48996491 | Crab aquaculture pond sediment |
| M13 | PRJNA1275853 | SAMN49014430 | Crab aquaculture pond sediment |
| M14 | PRJNA1275853 | SAMN49014431 | Crab aquaculture pond sediment |
| M15 | PRJNA1275853 | SAMN49014432 | Crab aquaculture pond sediment |
| M16 | PRJNA1275853 | SAMN49014433 | Crab aquaculture pond sediment |
| M17 | PRJNA1275853 | SAMN49014434 | Crab aquaculture pond sediment |
| M18 | PRJNA1275853 | SAMN49014435 | Crab aquaculture pond sediment |
| M19 | PRJNA1275853 | SAMN49014436 | Crab aquaculture pond sediment |
| M20 | PRJNA1275853 | SAMN49014437 | Crab aquaculture pond sediment |
| M21 | PRJNA1275853 | SAMN49014438 | Crab aquaculture pond sediment |
| S13 | PRJNA1275853 | SAMN49014457 | Crab aquaculture pond sediment |
| S14 | PRJNA1275853 | SAMN49014458 | Crab aquaculture pond sediment |
| S15 | PRJNA1275853 | SAMN49014459 | Crab aquaculture pond sediment |
| S16 | PRJNA1275853 | SAMN49014460 | Crab aquaculture pond sediment |
| S17 | PRJNA1275853 | SAMN49014461 | Crab aquaculture pond sediment |
| S18 | PRJNA1275853 | SAMN49014462 | Crab aquaculture pond sediment |
| S19 | PRJNA1275853 | SAMN49014463 | Crab aquaculture pond sediment |
| S20 | PRJNA1275853 | SAMN49014464 | Crab aquaculture pond sediment |
| S21 | PRJNA1275853 | SAMN49014465 | Crab aquaculture pond sediment |
| D13 | PRJNA1275853 | SAMN49014484 | Crab aquaculture pond sediment |
| D14 | PRJNA1275853 | SAMN49014485 | Crab aquaculture pond sediment |
| D15 | PRJNA1275853 | SAMN49014486 | Crab aquaculture pond sediment |
| D16 | PRJNA1275853 | SAMN49014487 | Crab aquaculture pond sediment |
| D17 | PRJNA1275853 | SAMN49014488 | Crab aquaculture pond sediment |
| D18 | PRJNA1275853 | SAMN49014489 | Crab aquaculture pond sediment |
| D19 | PRJNA1275853 | SAMN49014490 | Crab aquaculture pond sediment |
| D20 | PRJNA1275853 | SAMN49014491 | Crab aquaculture pond sediment |
| D21 | PRJNA1275853 | SAMN49014492 | Crab aquaculture pond sediment |

**Supplementary Table 2. Primers and qPCR protocols used in this study**

| **Target gene** | **Primers** | **Sequence (5’-3’)** | **Conditions** | **Reference** |
| --- | --- | --- | --- | --- |
| Bacterial 16S rRNA | 341F | CCTACGGGAGGCAGCAG | 95 °C for 1 min, 40 ×[95 °C for 15 s, 60 °C for 30 s, 72 °C for 30 s], 72 °C for 10 min | Muyzer et al., 1993 |
|  | 534R | ATTACCGCGGCTGCTGGCA |  |  |
| Archaeal 16S rRNA | Parch519F | CAGCCGCCGCGGTAA | 95 °C for 1 min, 40 ×[94 °C for 30 s, 57 °C for 40 s, 72 °C for 40 s], 72 °C for 10 min | Muyzer et al., 1993 |
|  | Arc915R | GTGCTCCCCCGCCAATTCCT |  |  |
| *pmoA* | Cmo182 | TCACGTTGACGCCGATCC | 95 °C for 1 min, 50 ×[95 °C for 30 s, 60 °C for 45 s, 72 °C for 40 s], 72 °C for 10 min | Niu et al., 2022 |
|  | Cmo568 | GCACATACTCCAATCCCCATC |  |  |
| *mcrA* | McrA159F | AAAGTGCGGAGCAGCAATCACC | 95 °C for 1 min, 50 ×[95 °C for 30 s, 62 °C for 45 s, 72 °C for 50 s], 72 °C for 10 min | Niu et al., 2022 |
|  | McrA345R | TCGTCCCATTCCTGCTGCATTGC |  |  |

The qPCR was performed using a 20 µl reaction system including: 10 µl of TB Green, 0.8 µl each of upstream and downstream primers, 1 µl of DNA template, and 7.4 µl of dd H_2_O.


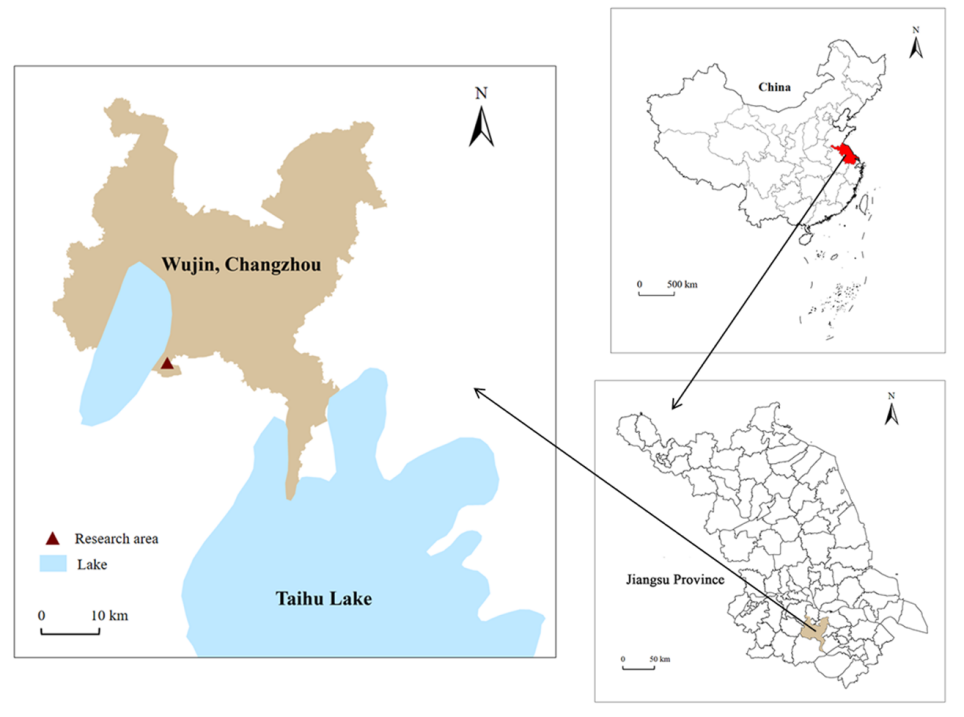


**Supplementary Figure 1. Distribution of sediment sampling sites (Jiangsu province, China) and distances to Taihu Lake.**

**
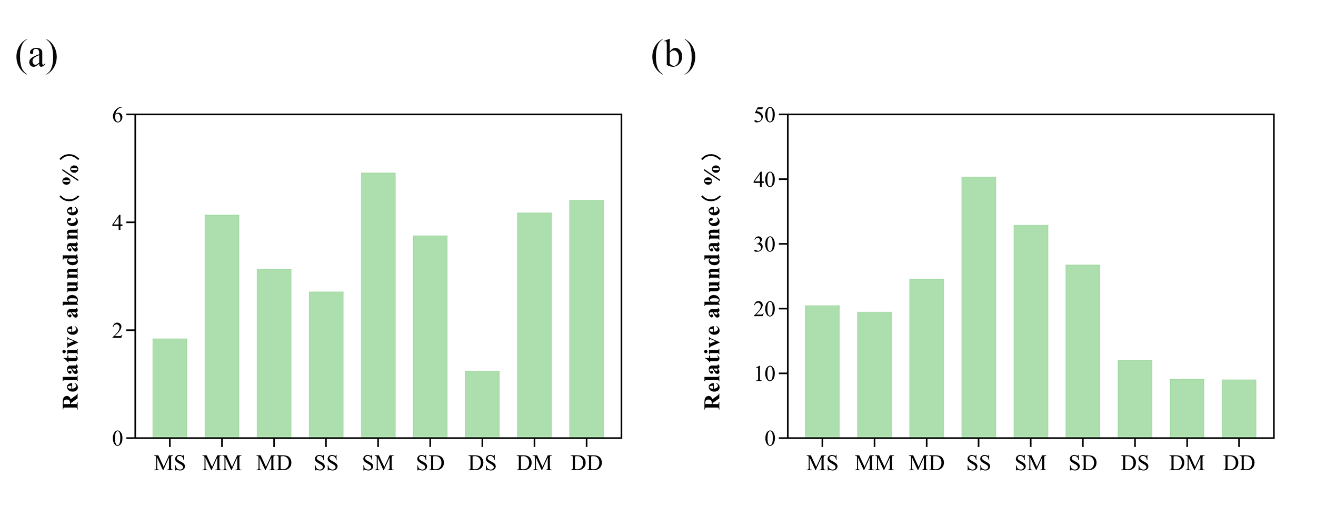
**

**Supplementary Figure 2. Total relative abundance of all methane-metabolizing microorganisms at the genus level.** (a) Methane-metabolizing bacterial communities. (b) Methane-metabolizing archaeal communities. MS: May 0−10 cm, MM: May 10−20 cm, MD: May 20−30 cm, SS: September 0−10 cm, SM: September 10−20 cm, SD: September 20−30 cm, DS: December 0−10 cm, DM: December 10−20 cm, DD: December 20−30 cm.

**Reference：**

Muyzer, G., Waal, E.C. de, Uitterlinden, A.G., (1993). Profiling of complex microbial populations by denaturing gradient gel electrophoresis analysis of polymerase chain reaction-amplified genes coding for 16S rRNA. Applied and Environmental Microbiology. https://doi.org/10.1128/aem.59.3.695-700.1993

Niu, Y., Zheng, Y., Hou, L., Gao, D., Chen, F., Pei, C., Dong, H., Liang, X., Liu, M., (2022). Microbial dynamics and activity of denitrifying anaerobic methane oxidizers in China’s estuarine and coastal wetlands. Science of The Total Environment 806, 150425. https://doi.org/10.1016/j.scitotenv.2021.150425
